# Dihydromyricetin alleviates ETEC K88-induced intestinal inflammatory injury by inhibiting quorum sensing-related virulence factors

**DOI:** 10.1186/s12866-025-03879-8

**Published:** 2025-04-09

**Authors:** Yaqian Shi, Jin Liu, Hualin Zhou, Zhongyuan Wu, Yinsheng Qiu, Chun Ye

**Affiliations:** 1 Hubei Key Laboratory of Animal Nutrition and Feed Science, School of Animal Science and Nutritional Engineering, Wuhan Polytechnic University, Wuhan, China; 2 Wuhan Engineering and Technology Research Center of Animal Disease-resistant Nutrition, School of Animal Science and Nutritional Engineering, Wuhan Polytechnic University, Wuhan, China; 3 Agricultural College, Xiangyang Polytechnic, Xiangyang, China

**Keywords:** Dihydromyricetin, ETEC, Quorum sensing, Virulence factor, Intestinal inflammation

## Abstract

**Background:**

Enterotoxigenic *Escherichia coli* (ETEC) is responsible for piglet diarrhea and causes substantial economic loss in the pig industry. Along with the restriction of antibiotics, natural compounds targeting bacterial virulence factors are supposed to be efficacious and attractive alternatives for controlling ETEC infection. This study aimed to investigate the influence of dihydromyricetin (DMY), a natural flavonoid compound, on the expression of virulence factors of ETEC and intestinal inflammatory injury.

**Results:**

DMY interfered with the quorum sensing (QS) of ETEC K88 since it decreased AI-2 secretion and downregulated the expression of LuxS and Pfs, which dominate AI-2 production, and decreased the expression mRNA level of genes (*lsrA*, *lsrB*, *lsrC*, *lsrD*, *lsrK*, and *lsrR*) that are involved in AI-2 internalization and signal transduction. Additionally, DMY markedly dampened the expression of QS-related virulence genes (*elt-1*, *estB*, *fliC*, *faeG*), biofilm formation, cell adhesion, and stress tolerance of ETEC K88. Furthermore, DMY treatment applied to the ETEC K88 infection in mice model resulted in decreased amount of heat-labile (LT) and heat-stable (ST) enterotoxins, reduced production of cAMP and cGMP, downregulated protein level of CFTR and upregulated expression of NHE3 in the ileum. In addition, the mRNA expression of proinflammatory cytokines (TNF-α, IL-1β, and IL-6) and histological damage in the ileum were significantly decreased by DMY treatment.

**Conclusions:**

DMY can inhibit the AI-2 QS and virulence factor expression, thereby attenuating the virulence of ETEC and alleviating intestinal inflammatory damage in ETEC K88-challenged mice. This study indicated that DMY has the potential to be a promising antivirulence agent for combating ETEC infection.

**Supplementary Information:**

The online version contains supplementary material available at 10.1186/s12866-025-03879-8.

## Introduction

Globally, diarrhea in piglets is one of the most challenging problems in swine production [1]. Although antibiotics are supposed to be the most effective therapeutic strategy for combating bacterial diseases, all currently available antibiotics are aimed at either killing the bacteria or inhibiting their growth, which puts strong selective pressure on bacterial communities. As a result, antibiotic resistance consistently appears and spreads rapidly, becoming a major barrier to controlling bacterial diseases. The use of antibiotics for animal production has been restricted worldwide. Thus, alternatives with different antibacterial mechanisms from antibiotics are desperately needed. Anti-virulence therapy focuses on blocking bacterial virulence factors and weakening bacterial pathogenesis, which puts less selective pressure on bacterial survival. It has been proven to be a promising strategy for combating bacteria diseases and slowing down the growing bacterial resistance to traditional antibiotics [2–7].

Enterotoxigenic *Escherichia coli* (ETEC) is one of the most common epidemical pathogens responsible for diarrhea in suckling and postweaning piglets and causes considerable economic loss in the pig industry [8]. The virulence and pathogenicity of ETEC are influenced by multiple factors, including adhesins, enterotoxins, biofilm formation and motility [9, 10]. F4 fimbriae (K88) are among the most prevalent adhesins and are composed of a major subunit encoded by *faeG* and several additional minor subunits [11]. Fimbrial adhesion to intestinal epithelial cells is thought to be the first and essential step enabling the colonization and proliferation of ETEC. Subsequently, ETEC secretes heat-labile (LT) and/or heat-stable (ST) enterotoxins, stimulating the accumulation of cyclic adenosine monophosphate (cAMP) and/or cyclic guanosine monophosphate (cGMP) in intestinal epithelial cells, followed by changes in the expression of diarrhea-related channels, including the cystic fibrosis transmembrane conductance regulator (CFTR) and Na^+^/H^+^-exchanger 3 (NHE3). Activation of CFTR and inhibition of NHE3 disturb intestinal chloride ion transport and Na^+^ absorption in intestinal cells, leading to diarrhea and mortality [12, 13]. Studies have indicated that the type I heat-labile enterotoxin LT-1 gene (*elt-1*), type I heat-stable enterotoxin STa gene (*estA*) and type II heat-stable enterotoxin STb gene (*estB*) are responsible for the pathogenesis of ETEC [14]. Additionally, flagellin has been implicated as a key virulence factor of *E. coli* required for biofilm formation, toxin delivery, adhesion and modulation of the immune response [15–17].

Quorum sensing (QS) plays critical roles in regulating virulence factors in many important pathogenic bacteria. QS is a process of bacterial chemical communication that relies on small signal molecules called autoinducers (AIs) in response to changes in population density. Various types of signalling molecules have been found to be produced by bacterial cells, among which autoinducer 2 (AI-2) is typically present in many gram-positive and gram-negative bacteria [18]. AI-2 is a byproduct of catalytic synthesis by the nucleosidase Pfs and the LuxS enzyme in an activated methyl cycle (AMC) [19]. Secreted AI-2 is transported into cells by the Lsr (LuxS-regulated) ABC transport system, which is encoded by the *lsr* operon (*lsrABCD*) and is subsequently phosphorylated by the kinase LsrK, followed by interaction with the *lsr* transcription repressor LsrR to switch on the *lsr* operon [20]. AI-2 signalling molecules that reach a certain concentration threshold are responsible for regulating a variety of physiological behaviors, including enterotoxin production, biofilm formation, stress resistance, motility, adhesion, drug resistance and bioluminescence [21, 22]. Therefore, the suppression of QS in ETEC is a promising anti-infection strategy without putting any selective pressure on the bacteria.

Numerous studies have indicated that plant-derived flavonoids such as baicalin [23], naringenin [24], quercetin [25], luteolin [26], and kaempferol [27] are able to inhibit QS-associated virulence factors to attenuate the virulence of pathogenic bacteria. Dihydromyricetin (DMY), also known as ampelopsin, is a natural flavonoid compound that is mainly purified from vine tea (*Ampelopsis grossedentata*) and has diverse biological activities, including anti-inflammatory, antitumour, antioxidant and antimicrobial activities [28]. Notably, DMY has potential inhibitory effects on the biofilm formation, adhesion and virulence genes expression in *Staphylococcus aureus* [29, 30]. However, scientific evidence is still lacking regarding whether DMY can suppress the QS, virulence factors and pathogenicity of ETEC. In view of this, this study investigated the inhibitory effects of DMY on QS, virulence genes expression, biofilm formation, pressure tolerance, and adhesion ability of ETEC in vitro and evaluated the protective effect against ETEC K88 infection in a mouse model. This study indicates the potential of DMY as a novel antivirulence drug for preventing and controlling ETEC infection.

## Materials and methods

### Reagents, bacterial strains and cell lines

Dihydromyricetin (DMY, purity ≥ 98%) was purchased from Chengdu Biopurify Phytochemicals Ltd. (Chengdu, China). DMY was dissolved in dimethyl sulfoxide (DMSO) (Sinopharm, Shanghai, China).

ETEC K88 and *E. coli* DH5α were cultured in Luria-Bertani (LB) media at 37 °C. *Vibrio harveyi* BB170, kindly provided by Professor Qigai He from Huazhong Agricultural University, China, was cultured in LB media at 30 °C.

Porcine intestinal epithelial cells (IPEC-1) were obtained from Professor Guoyao Wu, Texas A&M University, USA. The cells were cultured in Dulbecco’s Modified Eagle Medium/Nutrient Mixture F-12 (DME/F-12) (cytiva, USA) supplemented with 10% fetal ovine serum (Gibco, NY, USA), 1% penicillin-streptomycin (Gibco) and 1% glutamine (Solarbio, Nanjing, China) at 37 °C with 5% CO_2_.

### Antibacterial activity assays

The minimum inhibitory concentration (MIC) of DMY against ETEC K88 was determined by the broth dilution method. The ETEC K88 strain was grown to log-phase and diluted to 1 × 10^6^ colony-forming units (CFU)/mL in LB media. DMY was serially diluted twofold with DMSO, and each dilution was then diluted in LB broth (1:500). Subsequently, 100 µL of bacterial suspension mixed with an equal volume of DMY dilutions was added to a 96-well plate (Jet Biofil, Guangzhou, China). The final concentrations of DMY were 0, 25, 50, 100, 200, 400, 800, and 1600 µg/mL. Fresh LB media containing equivalent concentrations of DMY served as blank controls for subtracting background turbidity. The plate was incubated at 37 °C for 24 h, after which the absorbance at 600 nm (OD_600_) was measured using a microplate reader (SpectraMax i3x, Molecular Devices, China). The MIC here was identified as the lowest drug concentration that caused no visible growth in the LB media.

To verify the effect of DMY on the growth of ETEC K88, the growth curves of the bacteria were measured. Briefly, overnight-grown ETEC K88 cells were inoculated into 50 mL conical flasks containing 20 mL fresh LB broth supplemented with different concentrations of DMY (0, 50, 100, 200, or 400 µg/mL). After incubating for 0, 1, 2, 3, 4, 6, 8, 10, 12–24 h, the bacterial culture was serially diluted 10-fold in PBS, and the bacterial CFU in each sample were counted by plating the dilutions onto LB agar plates.

### AI-2 activity measurement

AI-2 activity was measured according to a previously described method [31]. Overnight-grown *V. harveyi* BB170 was diluted with fresh Autoinducer Bioassay (AB) medium at a ratio of 1:5000. DMY was diluted in LB media to concentrations of 50, 100, 200, and 400 µg/mL. Then, 180 µL of *V. harveyi* BB170 suspension mixed with 20 µL of diluted DMY was added to each well of a Corning^®^ 96-well black flat-bottomed polystyrene microplate. The plate was incubated at 30 ℃ for 5 h at 100 rpm. Bioluminescence was measured using a microplate reader (Molecular Devices) to estimate the effect of DMY on the AI-2 production of *V. harveyi* BB170. In addition, ETEC K88 cells were cultured in LB broth supplemented with different concentrations of DMY (0–400 µg/mL) for 4.5 h. The culture supernatants were collected by centrifugation and filtered through a 0.22 μm filter (Merck Millipore, Darmstadt, Germany). Similarly, the collected supernatants were mixed with a *V. harveyi* BB170 suspension at a ratio of 1:9 to assess whether DMY affects the secretion of AI-2 by ETEC K88. The culture supernatant of *E. coli* DH5α was used as the negative control.

### Protein levels of Pfs and LuxS

Western blotting analysis was used to evaluate the effect of DMY on the expression of Pfs and LuxS. The ETEC K88 cells treated with different concentrations of DMY (ranging from 0 to 400 µg/mL) were collected, washed twice with PBS, and lysed by ultrasonication. The supernatants were collected by centrifugation, and the concentrations of proteins in the supernatants were measured using an enhanced BCA protein assay kit (Beyotime, Shanghai, China). Subsequently, 20 µg of the proteins from each sample were separated using SDS-12.5%-PAGE and transferred to PVDF membranes (Merck Millipore, Cork, IreIand). The membranes were incubated with anti-Pfs or anti-LuxS antibodies followed by horseradish peroxidase (HRP)-conjugated goat anti-rabbit IgG. GAPDH (glyceraldehyde 3-phosphate dehydrogenase) gene was detected as an internal reference for the tested proteins. Antibody-antigen complexes were visualized using an enhanced chemiluminescence (ECL)kit (ABclonal, Wuhan, China) and a FluroChem E imaging system (Protein Simple, Shanghai, China). The relative band densities of the proteins were quantified using ImageJ software (National Institutes of Health).

### Expression of QS-related genes and virulence genes

Reverse transcriptase quantitative PCR (RT-qPCR) analysis was carried out to evaluate the effects of DMY on the expression of QS-related genes and virulence genes in ETEC K88. Overnight-cultured ETEC K88 cells were inoculated into LB broth (1:100 dilution) supplemented with different concentrations of DMY (ranging from 0 to 400 µg/mL) and incubated at 37 °C for 4.5 h at 180 rpm. RNA was extracted using a Bacterial RNA Extraction Kit (Vazyme, Nanjing, China). The extracted RNA was reverse transcribed into cDNA using the HiScript II 1st Strand cDNA Synthesis Kit (+ gDNA wiper) (Vazyme). The mRNA level of each gene was examined individually using Taq Pro Universal SYBR qPCR Master Mix (Vazyme) in an ABI PRISM 7500 Fast Real-time PCR System. The thermocycling conditions for qPCR were as follows: initial denaturation at 95 °C for 30 s; amplification of 40 cycles at 95 °C for 10 s, 60 °C for 34 s; melting curve analysis at 95 °C for 15 s, 60 °C for 1 min, followed by 1% increment from 60 °C to 95 °C. The reference gene *rpoA* served as the internal control gene. The relative mRNA expression levels of QS-related genes (*luxS*,* pfs*,* lsrA*,* lsrB*,* lsrC*,* lsrD*,* lsrK* and *lsrR*) and virulence genes (*elt-1*, *estB*, *fliC* and *feaG*) were calculated using the 2^−ΔΔCT^ method as previously described [32]. The primers used are listed in Additional file 1.

### Detection of biofilm formation

Bacterial biofilm formation was determined by the crystal violet staining method [33]. ETEC K88 cells were grown to the logarithmic phase and diluted to 1 × 10^6^ CFU/mL in LB media. The bacterial suspensions were added to a 96-well plate (200 µL in each well) and subsequently treated with different concentrations of DMY (0–400 µg/mL). Fresh LB medium served as the blank control. The plate was incubated at 37 °C for 48 h without shaking and then washed with sterile PBS to remove the unbound cells. Biofilms were fixed with 200 µL of 99% (vol/vol) methanol for 15 min, dried at room temperature, and then stained with 0.1% crystal violet for 10 min. The unbound dye was discarded, and the cells were then completely removed from the wells by rinsing with PBS. The crystal violet bound to the biofilm was dissolved in 200 µL of 33% glacial acetic acid for 10 min. The OD_590_ was measured to evaluate the formation of biofilms using a microplate reader.

### Cell viability

To determine whether DMY has a toxic effect on IPEC-1 cells, a CCK-8 assay was performed using a Cell Counting Kit-8 (Vazyme, Nanjing, China). Briefly, IPEC-1 cells were grown to a monolayer in a 96-well plate and treated with various concentrations of DMY (0–800 µg/mL) for 12 h. Then, 10 µL of CCK-8 solution was added to each well of the plate. After being incubated for 2 h, the OD_450_ was measured using a microplate reader.

### Adhesion assay

IPEC-1 monolayer cells were treated with different concentrations of DMY (ranging from 0 to 400 µg/mL) for 2 h in a 12-well plate (Corning, USA), followed by infection with ETEC K88 at a multiplicity of infection (MOI) of 1:100. The plate was then incubated at 37 °C with 5% CO_2_ for 2 h. The cells in each well were washed three times with PBS to remove the nonadherent bacteria. After washing, 1 mL of 0.2% Triton X-100 (Solarbio, Nanjing, China) was added to the wells, which were incubated for 5 min to lyse the cells. The serial dilutions of the lysates were plated onto LB agar plates to quantity the bacteria adhering to IPEC-1 cells.

### Stress resistance assays

Stress resistance assays were performed as previously described with some modifications [34–36]. ETEC K88 cells were cultured in LB broth supplemented with different concentrations of DMY (0–400 µg/mL) for 12 h and diluted to 10^6^ CFU/mL. For the thermal stress test, 1 mL of bacterial suspension was incubated at 48 °C in a water bath for 1 h. For the antioxidative stress test, the bacterial suspension was exposed to 20 mM H_2_O_2_ at 37 °C for 30 min. For the osmotic stress assay, the bacterial suspension was mixed with 3 M potassium chloride (KCl) (Sinopharm, Shanghai, China) at a ratio of 1:1 and incubated at 37 °C for 1 h. Untreated cells from each sample were incubated at 37 °C for 30 min or 1 h. These cells served as controls in each assay. The CFU of viable cells posttreatment were measured via plate counting. The stress resistance levels were calculated as follows: (stressed sample CFU/mL)/(control sample CFU/mL)×100%.

### Protective effect of DMY against ETEC-K88 infection in a mouse model

The animal experiment was performed in accordance with the animal welfare standards, complied with the guidelines of the Experimental Animal Welfare Ethics Committee, Chinese Association for Laboratory Animal Sciences, and was approved by the Institutional Animal Care and Use Committee of Wuhan Polytechnic University, China (approval number: WHPU-F20231106). A total of 60 4-week-old specific-pathogen-free (SPF) female Kunming mice were purchased from the Center or Disease Control and Prevention of Hubei Province (Wuhan, China). After a 7-day adaptation period, the mice were randomly divided into six groups, namely, a normal control group (CN), an ETEC K88-infected model group (K88), an enrofloxacin treatment group (ENR), and three DMY treatment groups (DMY1, DMY2, and DMY3), with 10 mice in each group. Mice were allowed to access feed and water ad libitum throughout the experiment but fasted for 12 h before ETEC-K88 infection. To induce diarrhea, mice, except those in the CN group, were intraperitoneally injected with 0.2 mL of the ETEC-K88 suspension (2 × 10^8^ CFU/mL), and mice in the CN group were injected with 0.2 mL of sterile PBS. Subsequently, mice in the ENR group were orally administered 200 µL of enrofloxacin (5 mg/kg); mice in the DMY1, DMY2, and DMY3 groups were orally administered DMY suspended in 0.5% sodium carboxymethylcellulose (CMC-Na) solution at doses of 25 mg/kg, 50 mg/kg and 100 mg/kg, respectively. Additionally, all the treatments were administered once again after 12 h. The other two groups were orally administered 200 µL of 0.5% CMC-Na solution per mouse. The body weight was monitored at 6 h intervals after infection for 24 h. At 24 h post infection, all the mice were deeply anesthetized by intramuscular injection of Zoletil^®^ 50 (Virbac, Carros, France) and then humanely euthanized by cervical dislocation. Ileum tissues were harvested, weighed and homogenized in PBS. The supernatants were collected by centrifugation for 10 min at 5000 × *g* and 4 °C. The concentrations of cAMP, cGMP, and *E. coli* LT and ST enterotoxins in the supernatants were measured with ELISA kits (Spbio, Wuhan, China) according to the manufacturer’s instructions.

To detect the protein levels of CFTR and NHE3, the protein concentration in the supernatant of the homogenized ileum tissue samples was quantified via an enhanced BCA protein assay kit (Beyotime); then, 20 µg of the protein from each sample was subjected to western blotting analysis as described above. Anti-CFTR polyclonal antibody (ABclonal, Wuhan, China), anti-NHE3 polyclonal antibody (ABclonal, Wuhan, China) and anti-β-actin polyclonal antibody (Proteintech Group, Wuhan, China) were used for the analysis.

For the inflammatory response assay, total RNA was extracted from the ileum tissues using RNA Isolator Total RNA Extraction Reagent (Vazyme, Nanjing, China). The extracted RNA was used to determine the mRNA levels of proinflammatory cytokines (TNF-α, IL-1β and IL-6) via RT-qPCR analysis as described above. β-Actin was used as the control gene for normalization. The primers used are listed in Additional file 1.

To evaluate intestinal morphology, ileum tissue samples were fixed in 4% paraformaldehyde, dehydrated, paraffin embedded, cut into slices, and stained with haematoxylin and eosin (H&E). The histological and morphological changes in the ileum were observed under an upright microscope (Olympus BX43, Japan).

### Statistical analysis

All the results of this study were statistically analysed using one-way ANOVA followed by Duncan’s post hoc test in SPSS 18.0 software (SPSS, Inc., Chicago, IL, USA). The data are presented as the mean ± standard deviation (SD) from at least three independent experiments. *p* < 0.05 and *p* < 0.01 were considered to indicate statistical significance.

## Results

### The antibacterial effect of DMY on ETEC K88

**Fig. 1:**
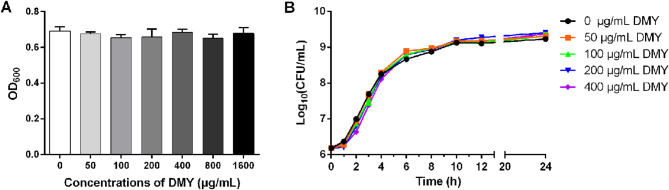
Antibacterial activity of DMY against ETEC K88. (**A**) MIC determination of DMY against ETEC K88. ETEC K88 cells were cultured with DMY (50–1600 µg/mL) at 37 °C for 24 h, after which the OD_600_ was measured. (**B**) Effect of DMY on the growth curves of ETEC K88 cells. ETEC K88 cells were cultured in LB broth supplemented with different concentrations of DMY (ranging from 0 to 400 µg/mL) at 37 °C and 180 rpm. After incubating for 1, 2, 3, 4, 6, 8, 10, 12 and 24 h, the number of CFUs in each sample was measured via the plate counting method. Data are shown as the mean ± standard deviation (SD)

The MIC of DMY and the growth curves of ETEC were measured to evaluate the antibacterial effect of DMY on ETEC. No obvious difference in growth was observed between the non-DMY-treated group and the DMY-treated group (50–1600 µg/mL) after 24 h of incubation at 37 °C, indicating that the MIC of DMY against ETEC K88 was greater than 1600 µg/mL (Fig. 1A). In addition, DMY (50–400 µg/mL) had no effect on the growth of the ETEC K88 cells (Fig. 1B).

### DMY interferes with the QS of ETEC K88

**Fig. 2:**
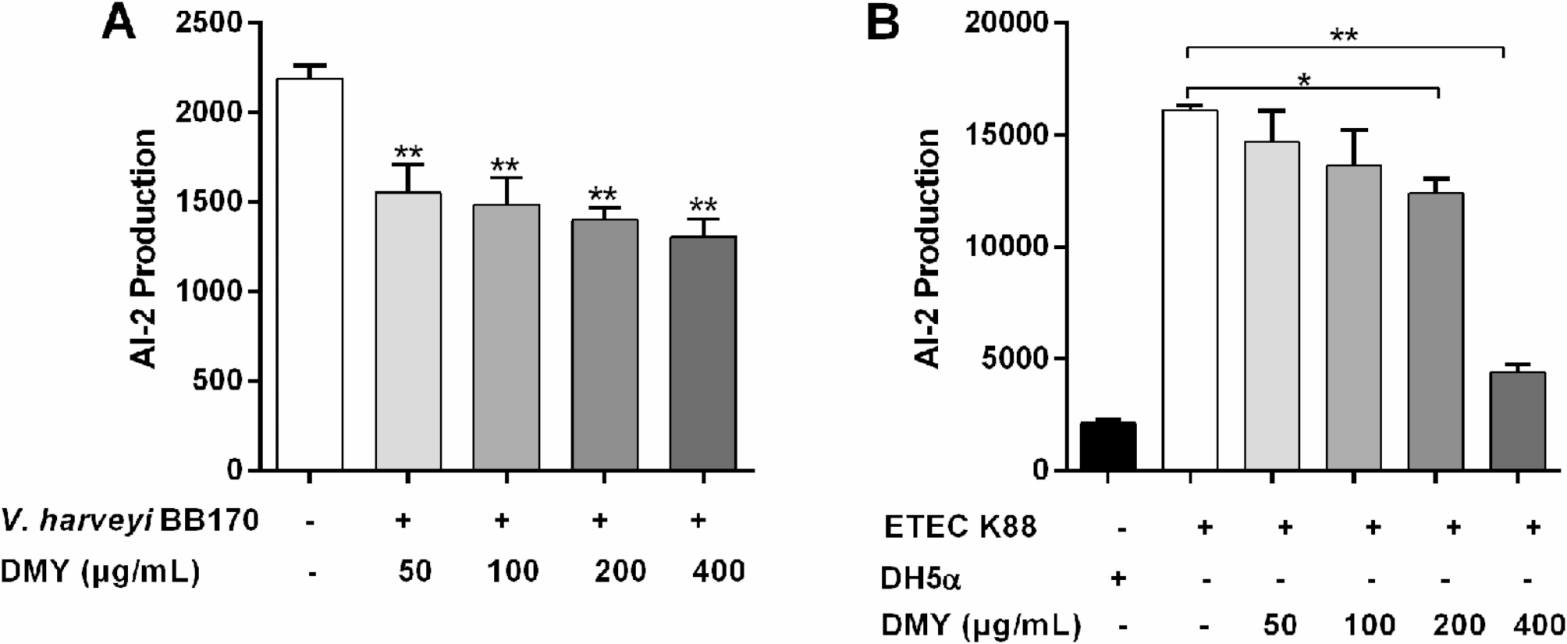
Measurement of AI-2 production. (**A**) Effects of DMY on AI-2 production by *V. harveyi* BB170. (**B**) Culture supernatants of ETEC K88 cells incubated with different concentrations of DMY (0–400 µg/mL) were collected, followed by mixing with the reporter strain *V. harveyi* BB170. After incubating for 5 h at 30 ℃, the AI-2 bioluminescence was measured. *E. coli* DH5α served as the negative control. Data are presented as the mean ± SD. *p < 0.05 or **p < 0.01 represents a significant difference between the groups with or without DMY treatment

**Fig. 3:**
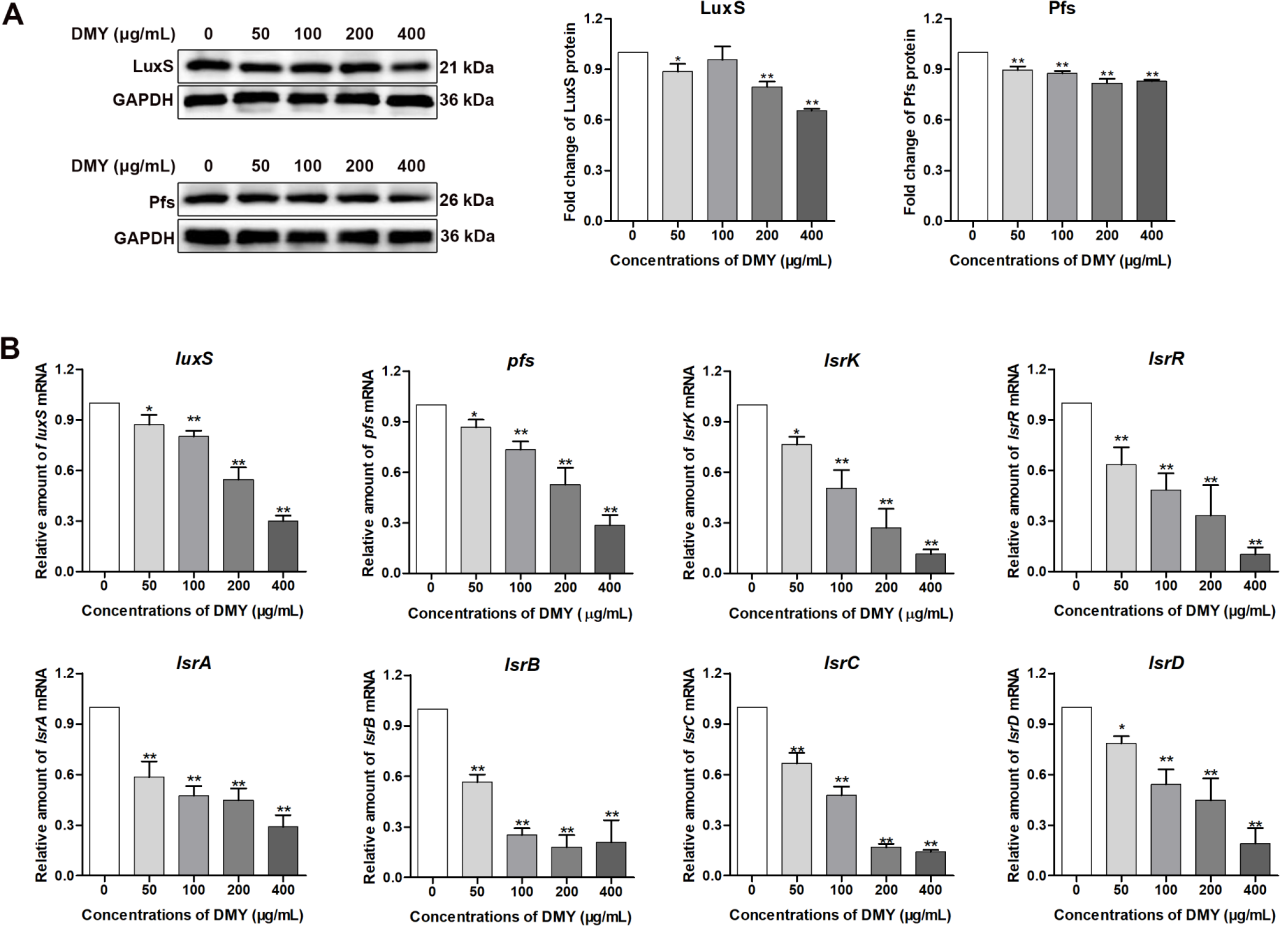
Effect of DMY on the expression of QS-related proteins and genes in ETEC K88 cells. (**A**) Effect of DMY on the expression of LuxS and Pfs proteins. The LuxS and Pfs proteins in ETEC K88 cells exposed to various concentrations of DMY (50, 100, 200, or 400 µg/mL) were detected via western blotting. The blots were cut prior to hybridization with antibodies. The fold changes in the proteins are given between the blots. The intensities of the LuxS and Pfs bands were measured and normalized to that of GAPDH. (**B**) Effect of DMY on the mRNA levels of QS-related genes. The relative mRNA levels of QS-related genes (*luxS*, *pfs*, *lsrK*, *lsrR*, *lsrA*, *lsrB*, *lsrC*, and *lsrD*) in ETEC K88 cells after treatment with various concentrations of DMY were determined via RT-qPCR. Data are shown as the mean ± SD. *p < 0.05, or **p < 0.01 represents a significant difference between the groups with or without DMY treatment

The effect of DMY on the QS of ETEC K88 was detected by measuring AI-2 production, the mRNA levels of QS-associated genes, and the protein levels of LuxS and Pfs. The effect of DMY on AI-2 production in *V. harveyi* BB170 and ETEC K88 was measured. As shown in Fig. 2, DMY (50–400 µg/mL) significantly suppressed the secretion of AI-2 by *V. harveyi* BB170. Considering that the amount of AI-2 produced by *V. harveyi* BB170 during growth was much lower than that produced by ETEC K88, DMY (200 and 400 µg/mL) significantly reduced AI-2 production compared with that of ETEC K88 without DMY even after eliminating the inhibitory effect of DMY on *V. harveyi* BB170. The protein levels of LuxS and Pfs in ETEC K88 cells decreased after treatment with DMY (Fig. 3A). Additionally, RT-qPCR analysis revealed that the expression of the QS-related genes (*luxS*,* pfs*,* lsrA*,* lsrB*,* lsrC*,* lsrD*,* lsrK* and *lsrR*) of ETEC K88 was downregulated by treatment with 50, 100, 200, or 400 µg/mL DMY in a dose-dependent manner (Fig. 3B). Taken together, these results suggested that DMY could interfere with the QS system of ETEC K88.

### DMY inhibits virulence genes expression in ETEC K88 cells

**Fig. 4:**
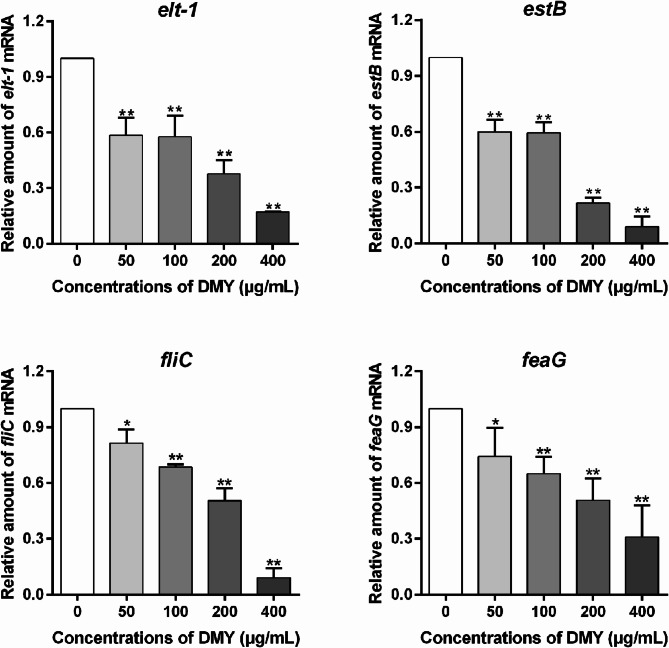
Effect of DMY on the expression of virulence genes in ETEC K88. ETEC K88 cells were treated with various concentrations of DMY (0, 50, 100, 200, or 400 µg/mL) for 4.5 h at 37 °C. The mRNA levels of *elt-1*, *estB*, *fliC*, and *feaG* were detected via qRT-PCR. Data are shown as the mean ± SD. *p < 0.05, or **p < 0.01 represents a significant difference between the groups with or without DMY treatment

As shown in Fig. 4, the RT-qPCR data indicated that the expression of the virulence genes of ETEC K88 including *elt-1*, *estB*, *fliC* and *feaG* decreased significantly after treatment with DMY (50, 100, 200, or 400 µg/mL) in a dose-dependent manner.

### DMY diminishes the biofilm formation of ETEC K88

**Fig. 5:**
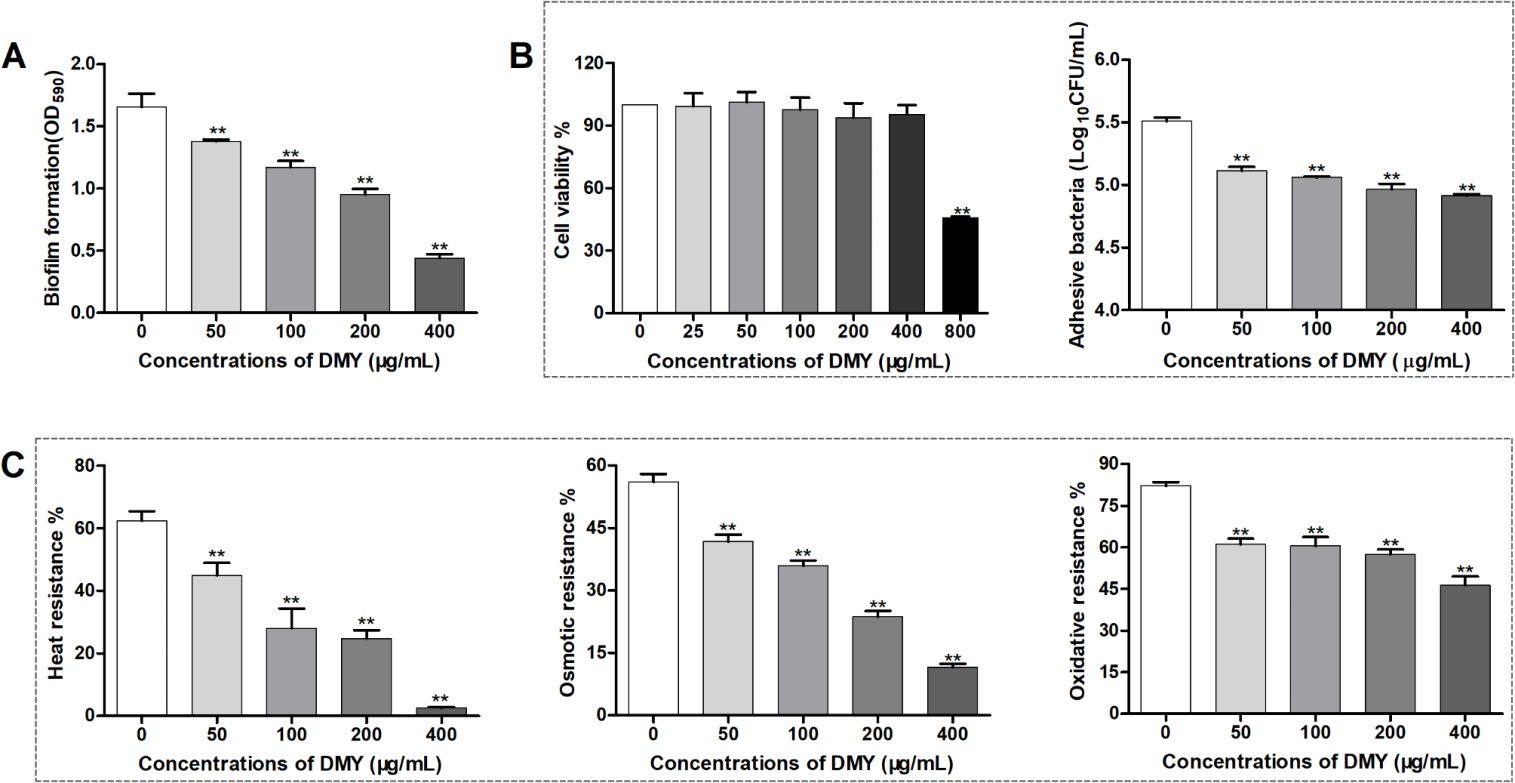
Effects of DMY on the biofilm formation, cell adhesion and stress resistance of ETEC K88. (**A**) ETEC K88 cells were grown in LB broth supplemented with various concentrations of DMY (0, 50, 100, 200, 400 µg/mL) for 48 h in a 96-well plate. The amount of biofilm formed was measured using a crystal violet staining method and is expressed as the OD_590_. (**B**) The cytotoxicity of DMY on IPEC-1 cells was detected using the CCK-8 assay. The IPEC-1 monolayer cells were treated with different concentrations of DMY (ranging from 0 to 400 µg/mL), followed by infection with ETEC K88 at a MOI of 1:100 for 2 h. The adhesion ability was expressed as the CFUs of bacteria adhering to IPEC-1 cells. (**C**) Effects of DMY on the stress resistance of ETEC K88 cells. ETEC K88 was grown in LB broth supplemented with different concentrations of DMY (0–400 µg/mL) for 12 h and diluted to 10^6^ CFU/mL. Heat stress resistance was measured by incubating the bacterial suspension at 48 °C for 1 h. Oxidative stress resistance was determined by exposing the bacterial suspension to 20 mM H_2_O_2_ at 37 °C for 30 min. Osmotic stress resistance was detected by mixing the bacterial suspension with an equal volume of 3 M KCl for 1 h. All the resistance levels were defined as the survival rate of ETEC K88 after treatment. Data are shown as the mean ± SD. **p < 0.01 represents a significant difference between the groups with or without DMY treatment

The effect of DMY on the biofilm formation of ETEC K88 was detected using the crystal violet staining method in a 96-well microtiter plate. The biofilm formation of ETEC K88 in the presence of DMY was considerably decreased in a dose-dependent manner compared to that in the absence of DMY (Fig. 5A), indicating that DMY was able to attenuate the biofilm formation of ETEC K88.

### DMY decreases the adhesion ability of ETEC K88 to IPEC-1 cells

As shown in Fig. 5B, the CCK-8 assay indicated that DMY concentrations less than 400 µg/mL were not toxic to the tested IPEC-1 cells, which had a viability greater than 90%. The adhesion of ETEC K88 to IPEC-1 cells was significantly lower in the presence of DMY (ranging from 50 to 400 µg/mL) than in the absence of DMY (*p* < 0.01).

### DMY treatment increases the sensitivity of ETEC K88 cells to stress conditions

The effects of DMY on the stress resistance of ETEC K88, including heat shock, oxidative stress and osmotic pressure, were detected and shown in Fig. 5C.

When the bacteria were incubated in a 48 ℃ water bath for 1 h, ETEC K88 cells pretreated with DMY at concentrations of 50, 100, 200 and 400 µg/mL exhibited survival rates of 44.98%, 28.05%, 24.77% and 2.57%, respectively, which were significantly lower than the 62.44% survival of ETEC K88 cells not treated with DMY (*p* < 0.01).

When the bacteria were exposed to 20 mM H_2_O_2_ for 30 min, the percentage of viable ETEC K88 cells pretreated with DMY at concentrations of 50, 100, 200 and 400 µg/mL (61.04%, 60.44%, 57.43% and 46.38%, respectively) decreased compared with that of non-DMY pretreated cells (82.12%).

Similarly, the resistance of ETEC K88 to osmotic pressure significantly decreased after pretreatment with DMY at concentrations of 50, 100, 200 and 400 µg/mL (*p* < 0.01), with 41.72%, 35.97%, 23.74%, and 11.55%, respectively, of the cells remaining viable compared with that of ETEC K88 without DMY treatment (56.12% survival).

Taken together, these data suggested that DMY treatment increased the sensitivity of ETEC K88 to various stress conditions.

### Protective effect of DMY against ETEC K88 challenge in a mouse model

**Fig. 6:**
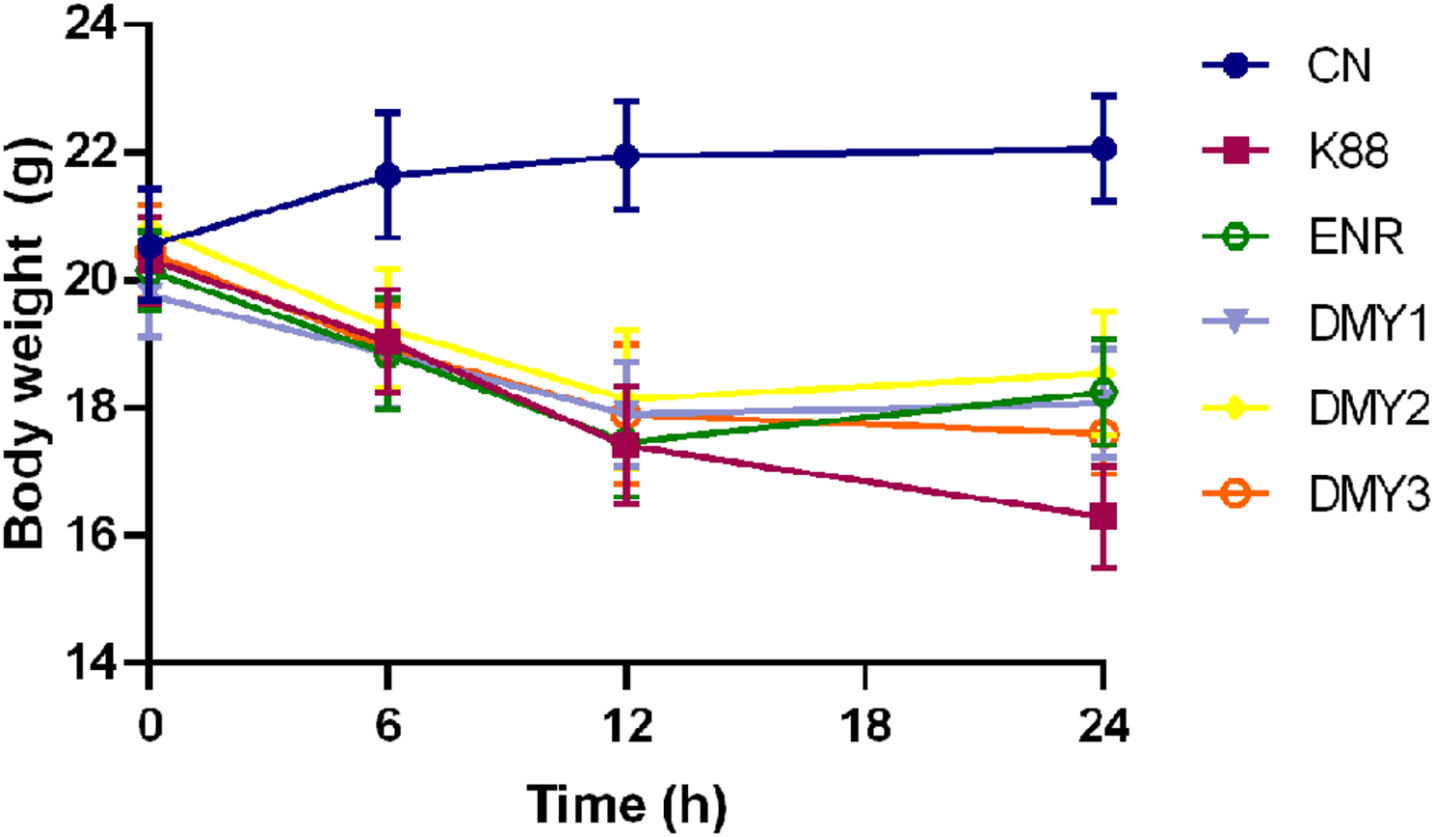
Body weights of the mice during the experimental period. Data are shown as the mean ± SD. CN, control group; K88, ETEC K88-challenged group; ENR, enrofloxacin treatment group; DMY1, DMY2 and DMY3, DMY treatment groups with DMY at doses of 25 mg/kg, 50 mg/kg and 100 mg/kg, respectively

The body weight of the mice gradually decreased within 24 h after ETEC K88 challenge, except for those in the control group. However, the body weight of the DMY-treated mice was slightly greater than that of the ETEC K88-infected mice 12 h after infection (Fig. 6).

**Fig. 7:**
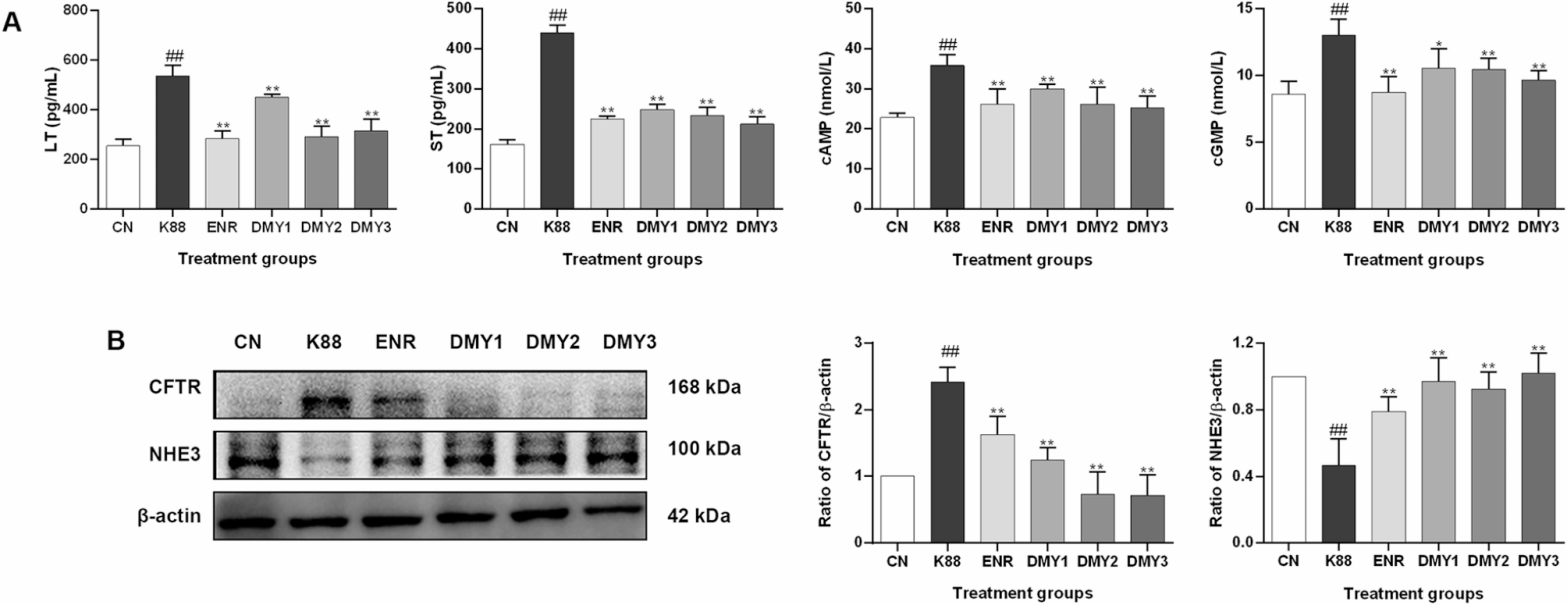
Effects of DMY on diarrhea-related factors. (**A**) The amounts of *E. coli* LT and ST enterotoxins, cAMP, and cGMP in the ileum were measured via ELISA. (**B**) The expression levels of CFTR and NHE3 proteins were determined via western blotting. The intensity of each protein band was quantified using ImageJ software. Data are shown as the mean ± SD. ^##^*p* < 0.01 vs. CN, *p < 0.05 vs. K88, **p < 0.01 vs. K88

The effects of DMY on enterotoxin production, cAMP and cGMP accumulation, and diarrhea-related channel (CFTR and NHE3) expression in mice were shown in Fig. 7. LT and ST enterotoxins play pivotal roles in the occurrence of diarrhea induced by ETEC. As shown in Fig. 7A, ETEC K88 infection resulted in significant increases in LT and ST in the ileum compared with those in the control group (*p* < 0.01). Treatment with DMY significantly decreased the amount of LT and ST in the ileum compared with that in the ETEC K88-infected group (*p* < 0.01). Enterotoxins usually cause the accumulation of cAMP and/or cGMP, followed by the activation of CFTR and the suppression of NHE3 [12, 13]. As expected, the amounts of cAMP and cGMP significantly increased in the ileum of mice after ETEC K88 infection, while DMY treatment significantly attenuated this increase (*p* < 0.01) (Fig. 7A). Similarly, the protein level of CFTR in the ileum was upregulated after ETEC K88 infection compared to that in the control group (*p* < 0.01). DMY significantly decreased the expression of CFTR (*p* < 0.01). Furthermore, the change in the Na^+^/H^+^ exchanger (NHE3) in the ileum completely reversed the change in CFTR. In brief, the expression of NHE3 in the ileum was lower in the ETEC K88-challenged group than in the control group (*p* < 0.01), while NHE3 expression was significantly reversed after DMY treatment (*p* < 0.01) (Fig. 7B).

**Fig. 8:**
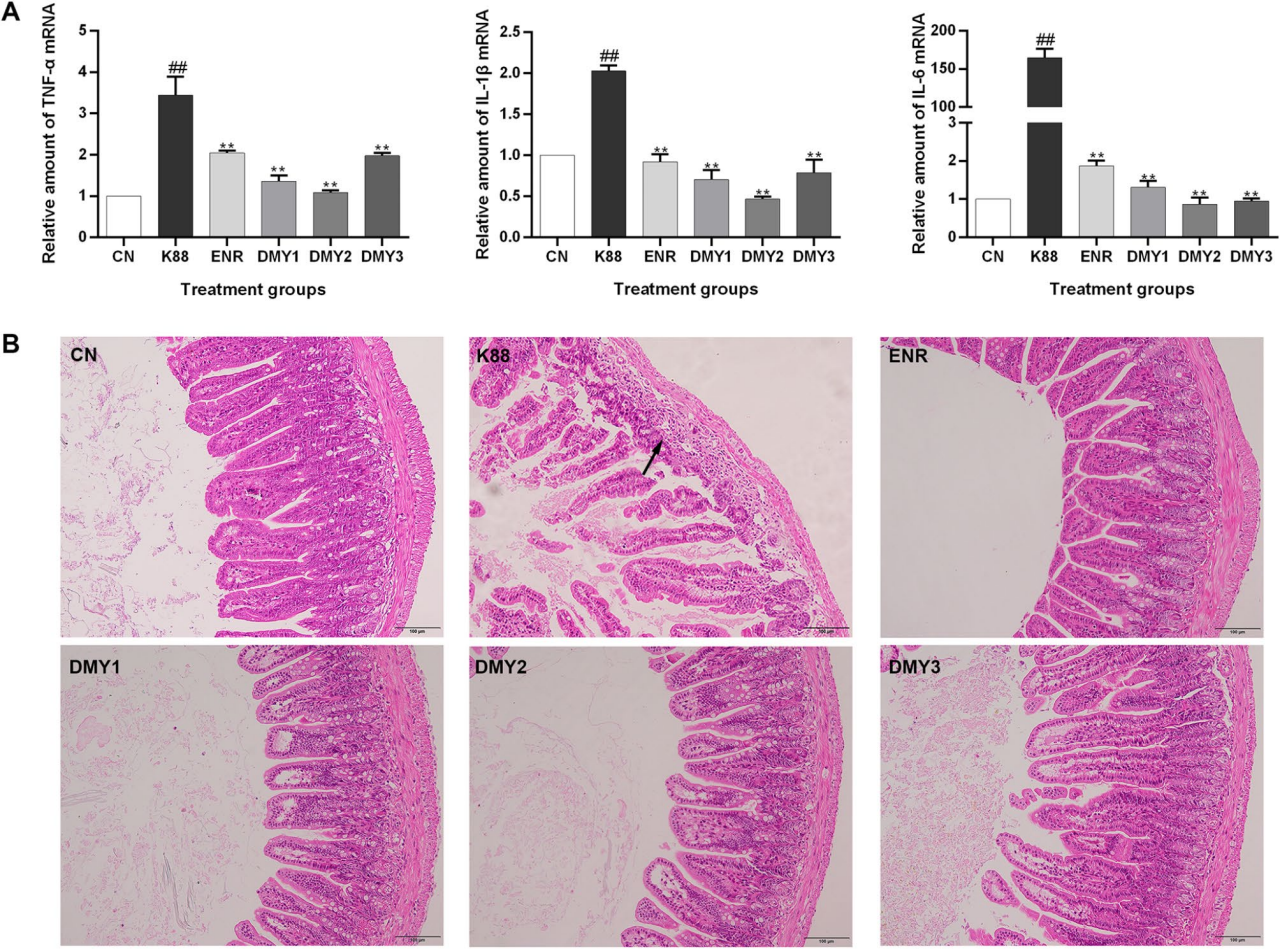
Effects of DMY on intestinal inflammation in mice. (**A**) Relative mRNA expression of inflammatory cytokines (TNF-α, IL-1β, and IL-6) in the ileum. Data are shown as the mean ± SD. ^##^*p* < 0.01 vs. CN, **p < 0.01 vs. K88. (**B**) Comparison of microscopic pathological changes in the ileum. Arrow represents inflammation cell infiltration. Histopathology was performed using an upright microscope at 200× magnification

The mRNA levels of proinflammatory cytokines (TNF-α, IL-1β and IL-6) in the ileum tissues were upregulated after ETEC K88 challenge relative to those in the control group (*p* < 0.01). However, treatment with DMY protected mice against the intestinal inflammatory responses induced by ETEC challenge, as indicated by the decreased expression levels of ileal TNF-α, IL-1β and IL-6 in the DMY treatment groups (*p* < 0.01) (Fig. 8A).

Ileal morphology determined via H&E staining was used to evaluate the improvement effect of DMY on the intestinal inflammatory injury caused by ETEC K88. Compared with those in the control group, inflammatory cell aggregation was observed in the ileum of the ETEC K88-challenged mice. However, the histopathological damage in the ileum of mice in the DMY treatment groups was almost completely reversed (Fig. 8B). Additionally, all the findings of DMY treatment were comparable to those of enrofloxacin treatment.

## Discussion

ETEC is a major cause of diarrhea in piglets, posing a significant threat and challenge to the swine industry. To slow the development of antibiotic resistance, the inhibition of virulence factors or virulence-associated processes with natural compounds has been considered a novel alternative therapeutic strategy to combat bacteria-mediated diseases [37, 38]. Therefore, this work explored the effects of the flavonoid compound DMY on the QS and virulence factor expression of ETEC K88, as well as its protective effect against ETEC K88 infection. Notably, DMY had no antibacterial effect on ETEC K88 with a MIC exceeding 1600 µg/mL, and did not affect bacterial growth. Nevertheless, DMY interfered with the AI-2 QS system, repressed virulence genes expression, prevented biofilm formation and cell adhesion, and decreased stress tolerance. Furthermore, DMY protected ETEC K88-challenged mice from intestinal inflammatory injury by inhibiting enterotoxin production and regulating the expression of diarrhea-related channels.

QS is essential for the pathogenicity of many pathogenic bacteria [21, 22] and is considered a key target for screening alternative therapeutic drugs to combat bacterial infection. AI-2, which mediates QS, is considered to be a universal signalling molecule involved in intra- and interspecies bacterial communication [39]. LuxS and Pfs are significant enzymes involved in AI-2 synthesis via AMC. The deletion of the *luxS* and *pfs* genes in *E. coli* abolished the production of AI-2, reduced adhesion ability, decreased biofilm formation and motility, downregulated the transcript levels of virulence genes, and attenuated pathogenicity [40–42]. Additionally, the uptake of AI-2 in *E. coli* relies on the Lsr ABC transport system. The periplasmic protein LsrB serves as an AI-2 receptor responsible for the internalization of AI-2 in *E. coli* along with other Lsr transporter proteins (LsrA, LsrC, LsrD). Once inside the cell, accumulated AI-2 is phosphorylated by the kinase LsrK, followed by binding to LsrR to initiate transport by the *lsr* operon [20]. Likewise, *lsrR*, *lsrK*, and *lsrB*, as well as *lsr* operon deletion mutants, have been shown to cause impaired AI-2 production, resulting in reduced expression of AI-2-regulated virulence genes [43–45]. In this study, we found that AI-2 production of ETEC K88 cells exposed to DMY was diminished. Additionally, the expression of *luxS* and *pfs* was significantly reduced in response to DMY at both the mRNA and protein levels. Furthermore, DMY significantly decreased the expression of *lsrA*, *lsrB*, *lsrC*, *lsrD*, *lsrK*, and *lsrR*. The present results suggested that DMY could suppress the synthesis and internalization of AI-2 and consequently interfere with QS in ETEC K88 cells.

Pathogenic ETEC requires various virulence factors to cause piglet diarrhea. QS has been proposed to regulate multiple biological activities, such as virulence genes expression, adhesion, biofilm formation and environmental adaptability. Unsurprisingly, the virulence genes (*faeG*, *fliC*, *elt-1* and *estB*) and virulence-associated traits of ETEC were inhibited by DMY as a result of QS suppression. Biofilms, surface-associated aggregations of microbial communities enveloped by extracellular matrix components, are believed to play critical roles in bacterial virulence, antimicrobial resistance, evasion of the host immune system and countering harsh environments [46]. Many studies have shown a positive correlation between AI-2 production and biofilm formation [47, 48], which is also associated with cell adhesion [49]. Adherence to intestinal epithelial cells mediated by adhesins is the first step for ETEC to trigger an infection. The most important adhesin in ETEC K88 is F4 fimbriae. FaeG is a major subunit of F4 fimbriae that is conducive to the attachment of pathogenic bacteria to host cells. The *faeG* mutant of ETEC displayed a significant reduction in adhesion to intestinal epithelial cells [50] and attenuated intestinal mucosal damage and apoptosis in piglets [51]. Additionally, the major flagellin protein FliC is also supposed to be an important virulence factor. The deletion of *fliC* reduced the adhesion ability of ETEC to porcine intestinal epithelial cells (IPEC-J2), downregulated F4 fimbriae expression, and prevented biofilm formation [16, 17]. Here, we found that DMY could inhibit biofilm formation and protect IPEC-1 cells from adhesion by ETEC, which was likely attributed to DMY inhibiting the expression of *feaG* and *fliC*. Furthermore, the main transmission route of ETEC infection was fecal-oral transmission. Bacterial biofilm formation contributes to long-term survival after ETEC is discharged into the environment through feces and is crucial for bacterial adaptability to environmental changes as well as successful colonization in the intestinal tract [52]. As expected, our study indicated that ETEC K88 was more sensitive to heat shock, oxidative stress, and osmotic pressure after exposure to DMY, suggesting that DMY negatively regulates bacterial survival in response to environmental stresses.

Once ETEC cells adhere to and colonize intestinal epithelial cells, LT and/or ST enterotoxins are secreted by ETEC and play critical roles in triggering ETEC pathogenesis and diarrhea occurrence. To determine whether DMY attenuates the pathogenicity of ETEC by inhibiting virulence factors, an ETEC K88 challenge model was used to investigate the levels of enterotoxins and diarrhea-related channels in mice. As expected, DMY weakened the adverse effect on body weight in mice challenged with ETEC K88. The amounts of both LT and ST enterotoxins increased in the ileum of mice infected with ETEC K88, while DMY treatment decreased the enterotoxin levels. This difference might be attributed to DMY inhibiting the expression of enterotoxin-encoding genes (*elt-1* and *estB*) of ETEC K88. The occurrence of diarrhea is mainly due to abnormal transportation of electrolytes and water. LT and ST enterotoxins produced by ETEC result in the continuous accumulation of cAMP and cGMP, which activate the ion channel CFTR and inhibit the sodium channel NHE3, ultimately causing increased chloride ion and water secretion and reduced sodium absorption. *E. coli* enterotoxins are unable to stimulate fluid secretion in the absence of functional CFTR [13]. Studies have shown increased CFTR and decreased NHE3 in the ileum of diarrheal piglets challenged with ETEC K88 compared with those in the non-ETEC K88 challenge group [53, 54]. In the present study, DMY treatment significantly inhibited the increase in cAMP and cGMP in the ileum mediated by ETEC. Additionally, compared with those in the control group, the protein abundance of CFTR and the level of NHE3 in the ileum were greater in the ETEC K88-infected group, while DMY significantly reversed this trend. The above results suggested that DMY treatment might be able to effectively alleviate ETEC-K88-induced diarrhea via the potential mechanism of reducing enterotoxin production and regulating the expression of CFTR and NHE3 in the small intestine to decrease intestinal fluid secretion. Furthermore, LT enterotoxin is supposed to enhance ETEC adherence to intestinal epithelial cells [55]; however, whether the decrease in the adhesion ability of ETEC to DMY is related to reduced enterotoxins needs further research.

ETEC cells established in the small intestine usually trigger an immune response that plays critical roles in the defence against pathogenic infection. The production and regulation of proinflammatory cytokines such as TNF-α, IL-6, and IL-1β are strongly implicated in host defence in response to ETEC infection, while overexpression of these cytokines results in reverse pathological effects that lead to inflammation [56]. Studies have indicated that ETEC infection significantly increases the levels of proinflammatory cytokines (TNF-α, IL-1β and IL-6) in the jejunum of mice [57]. Consistent with these findings, ETEC infection significantly stimulated the expression of TNF-α, IL-1β and IL-6 in the ileum of mice, while DMY significantly downregulated the expression of TNF-α, IL-1β and IL-6. Notably, DMY was able to diminish inflammatory cell infiltration in ileum tissues from mice infected with ETEC K88. These results suggested that DMY could alleviate ETEC K88 intestinal inflammatory injury, which is consistent with a previous study in a weaned piglet model [58]. Additionally, studies have indicated that ST and LT enterotoxins are related to inflammatory immune responses and ETEC enterotoxins and can enhance the secretion of proinflammatory cytokines, such as IL-1β, IL-6, IL-8, and TNF-α, in the small intestine [55, 59, 60]. We speculated that DMY alleviated ETEC K88-induced intestinal inflammation by restricting enterotoxins production. In summary, our findings shed light on the protective effect of DMY on ETEC-K88 infection. Regretfully, only female mice were included in our present study. Whether the protection by DMY against ETEC-K88 infection was a sex-specific effect is still unclear, which needs our further study.

## Conclusions

The results of this study revealed that DMY suppressed the expression of virulence genes and virulence-associated traits of ETEC K88 by interfering with AI-2 QS. As a consequence, DMY inhibited enterotoxins release and conferred protection against ETEC K88-induced intestinal inflammatory injury in a mouse model. This study suggested that DMY has potential as a promising antivirulence agent in the fight against ETEC K88 infection.

## Electronic supplementary material

Below is the link to the electronic supplementary material.

Supplementary Material 1

Supplementary Material 2

## Data Availability

Data is provided within the manuscript or supplementary information files.

## Abbreviations

DMY: Dihydromyricetin
ETEC: Enterotoxigenic Escherichia coli
QS: Quorum sensing
LT: Heat-labile enterotoxin
ST: Heat-stable enterotoxin
cAMP: Cyclic adenosine monophosphate
cGMP: Cyclic guanosine monophosphate
CFTR: Cystic fibrosis transmembrane conductance regulator
NHE3: Na^+^/H^+^-exchanger 3
AMC: Activated methyl cycle
Lsr: LuxS-regulated
