# Supplementary material for: Dihydromyricetin alleviates ETEC K88-induced intestinal inflammatory injury by inhibiting quorum sensing-related virulence factors
Source: BMC Microbiol. 2025 Apr 9;25:201. doi: 10.1186/s12866-025-03879-8 (PMC11980137; doi:10.1186/s12866-025-03879-8)
Supplement: Supplementary file 1 — Supplementary Material 1 [file 12866_2025_3879_MOESM1_ESM.docx]

Additional file 1. List of primers used in this study

| **Primers** | **Orientation** | **Sequences (5’-3’)** |
| --- | --- | --- |
| *luxS* | Forward | CAGTGCCAGTTCTTCGTTGC |
|  | Reverse | TGAACGTCTACCAGTGTGGC |
| *pfs* | Forward | CTGTTGGAACACTGCAAGCC |
|  | Reverse | GCGTCGTGATAACGTGCTTC |
| *lsrA* | Forward | CAGCTTGTTCCGGTTGGTTG |
|  | Reverse | TTATCTGCCGGAAGATCGCC |
| *lsrB* | Forward | TTTCTGGTCAGGTACAGTTG |
|  | Reverse | TAATGTAGTAAGAGCGGCAC |
| *lsrC* | Forward | CTGTTGGGGATGTTACTGAA |
|  | Reverse | ACAATGGCAGGGATCTTTAG |
| *lsrD* | Forward | GTGACTTTATCTGCATTGGC |
|  | Reverse | AGCAGTAAGGTCAGGAGTAT |
| *lsrK* | Forward | GTTTAGGCATCGACACCTAT |
|  | Reverse | GATACCAGGTTTTAAAGCGC |
| *lsrR* | Forward | GAACAGTTAATGATTGGCCG |
|  | Reverse | GCTTCGGCTTTATTTTCTCC |
| *elt-1* | Forward | CGTTCCGGAGGTCTTATGCC |
|  | Reverse | GGTTTGTGTTCCTCTCCGCGT |
| *estB* | Forward | CAATAGCATTCAGCACCATA |
|  | Reverse | AATGTCCGTCTTGCGTTA |
| *fliC* | Forward | AACGGTTAGCAATCGCCTGA |
|  | Reverse | GCCTCTCGCTGATCACTCAA |
| *feaG* | Forward | CTTTTTGCCGACGGGTTGAG |
|  | Reverse | AACCTGCGACGTCAACAAGA |
| *rpoA* | Forward | GCACCAAAGAAGGCGTTCAG |
|  | Reverse | ATATCGGCTGCAGTCACAGG |
| TNF-α | Forward | GACCCTCACA CTCAGATCATCTT |
|  | Reverse | CCTTGAAGAGAACCTGGGAGTAG |
| IL-1β | Forward | GCAGAGCACAAGCCTGTCTTCC |
|  | Reverse | ACCTGTCTTGGCCGAGGACTAAG |
| IL-6 | Forward | TCTGCTCTGGAGCCCACCAAG |
|  | Reverse | CCAGCATCAGTCCCAAGAAGGC |
| β-actin | Forward | GGCTGTATTCCCCTCCATCG |
