# Supplementary material for: Dihydromyricetin alleviates ETEC K88-induced intestinal inflammatory injury by inhibiting quorum sensing-related virulence factors
Source: BMC Microbiol. 2025 Apr 9;25:201. doi: 10.1186/s12866-025-03879-8 (PMC11980137; doi:10.1186/s12866-025-03879-8)
Supplement: Supplementary file 2 — Supplementary Material 2 [file 12866_2025_3879_MOESM2_ESM.pptx]

## Slide 1
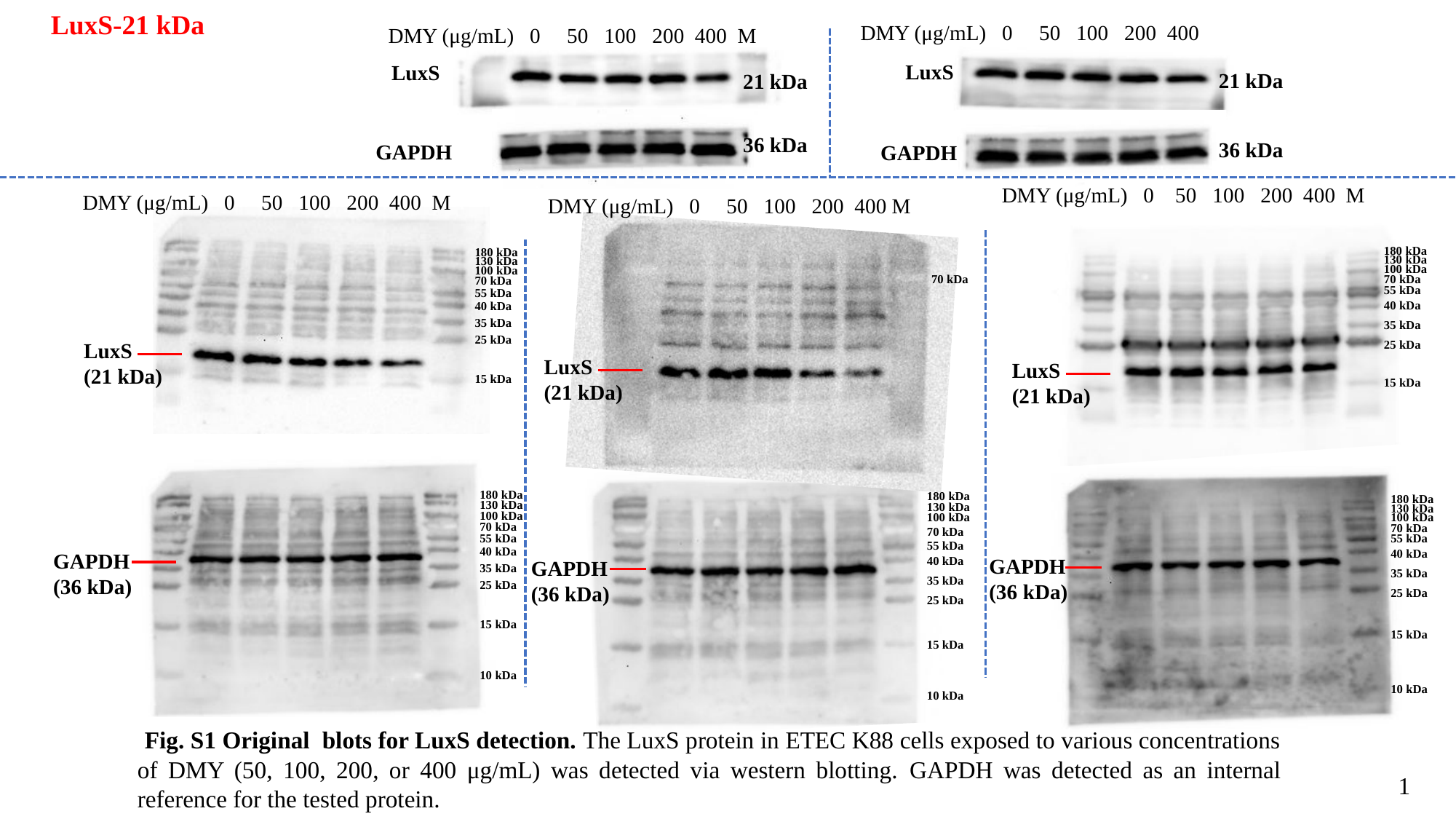

LuxS-21 kDa
DMY (μg/mL) 0 50 100 200 400
DMY (μg/mL) 0 50 100 200 400 M
LuxS
LuxS
 21 kDa
 21 kDa
GAPDH
GAPDH
 36 kDa
 36 kDa
DMY (μg/mL) 0 50 100 200 400 M
DMY (μg/mL) 0 50 100 200 400 M
DMY (μg/mL) 0 50 100 200 400 M
180 kDa
130 kDa
100 kDa
70 kDa
55 kDa
40 kDa
35 kDa
25 kDa
15 kDa
180 kDa
130 kDa
100 kDa
70 kDa
55 kDa
40 kDa
35 kDa
25 kDa
15 kDa
70 kDa
LuxS
(21 kDa)
LuxS
(21 kDa)
LuxS
(21 kDa)
180 kDa
130 kDa
100 kDa
70 kDa
55 kDa
40 kDa
35 kDa
25 kDa
15 kDa
10 kDa
180 kDa
130 kDa
100 kDa
70 kDa
55 kDa
40 kDa
35 kDa
25 kDa
15 kDa
10 kDa
180 kDa
130 kDa
100 kDa
70 kDa
55 kDa
40 kDa
35 kDa
25 kDa
15 kDa
10 kDa
GAPDH
(36 kDa)
GAPDH
(36 kDa)
GAPDH
(36 kDa)
 Fig. S1 Original blots for LuxS detection. The LuxS protein in ETEC K88 cells exposed to various concentrations of DMY (50, 100, 200, or 400 μg/mL) was detected via western blotting. GAPDH was detected as an internal reference for the tested protein.
1

## Slide 2
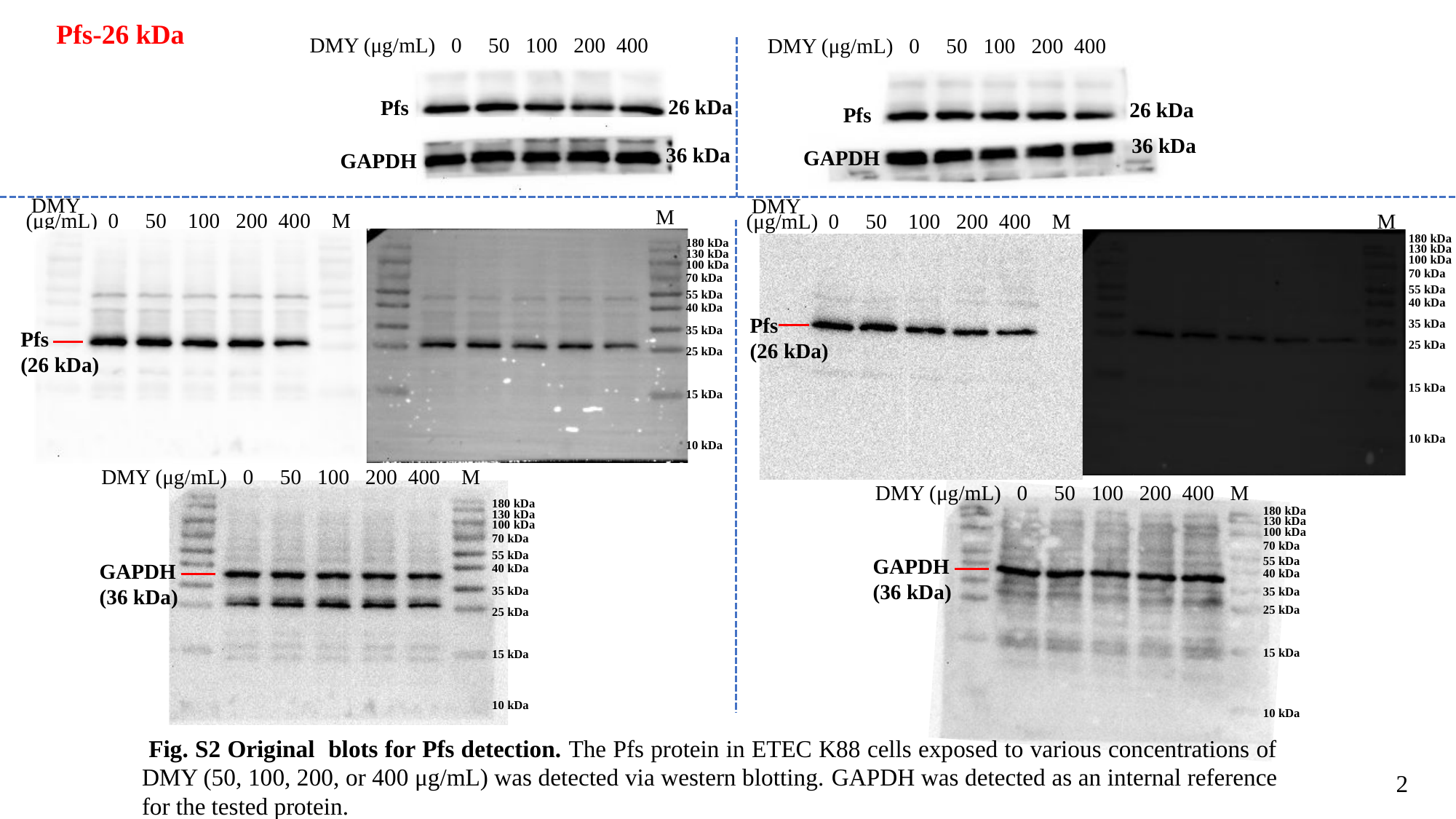

Pfs-26 kDa
DMY (μg/mL) 0 50 100 200 400
DMY (μg/mL) 0 50 100 200 400
 26 kDa
Pfs
 26 kDa
Pfs
 36 kDa
 36 kDa
GAPDH
GAPDH
 DMY
 (μg/mL) 0 50 100 200 400 M
 DMY
 (μg/mL) 0 50 100 200 400 M
M
M
180 kDa
130 kDa
100 kDa
70 kDa
55 kDa
40 kDa
35 kDa
25 kDa
15 kDa
10 kDa
180 kDa
130 kDa
100 kDa
70 kDa
55 kDa
40 kDa
35 kDa
25 kDa
15 kDa
10 kDa
Pfs
(26 kDa)
Pfs
(26 kDa)
DMY (μg/mL) 0 50 100 200 400 M
DMY (μg/mL) 0 50 100 200 400 M
180 kDa
130 kDa
100 kDa
70 kDa
55 kDa
40 kDa
35 kDa
25 kDa
15 kDa
10 kDa
180 kDa
130 kDa
100 kDa
70 kDa
55 kDa
40 kDa
35 kDa
25 kDa
15 kDa
10 kDa
GAPDH
(36 kDa)
GAPDH
(36 kDa)
 Fig. S2 Original blots for Pfs detection. The Pfs protein in ETEC K88 cells exposed to various concentrations of DMY (50, 100, 200, or 400 μg/mL) was detected via western blotting. GAPDH was detected as an internal reference for the tested protein.
2

## Slide 3
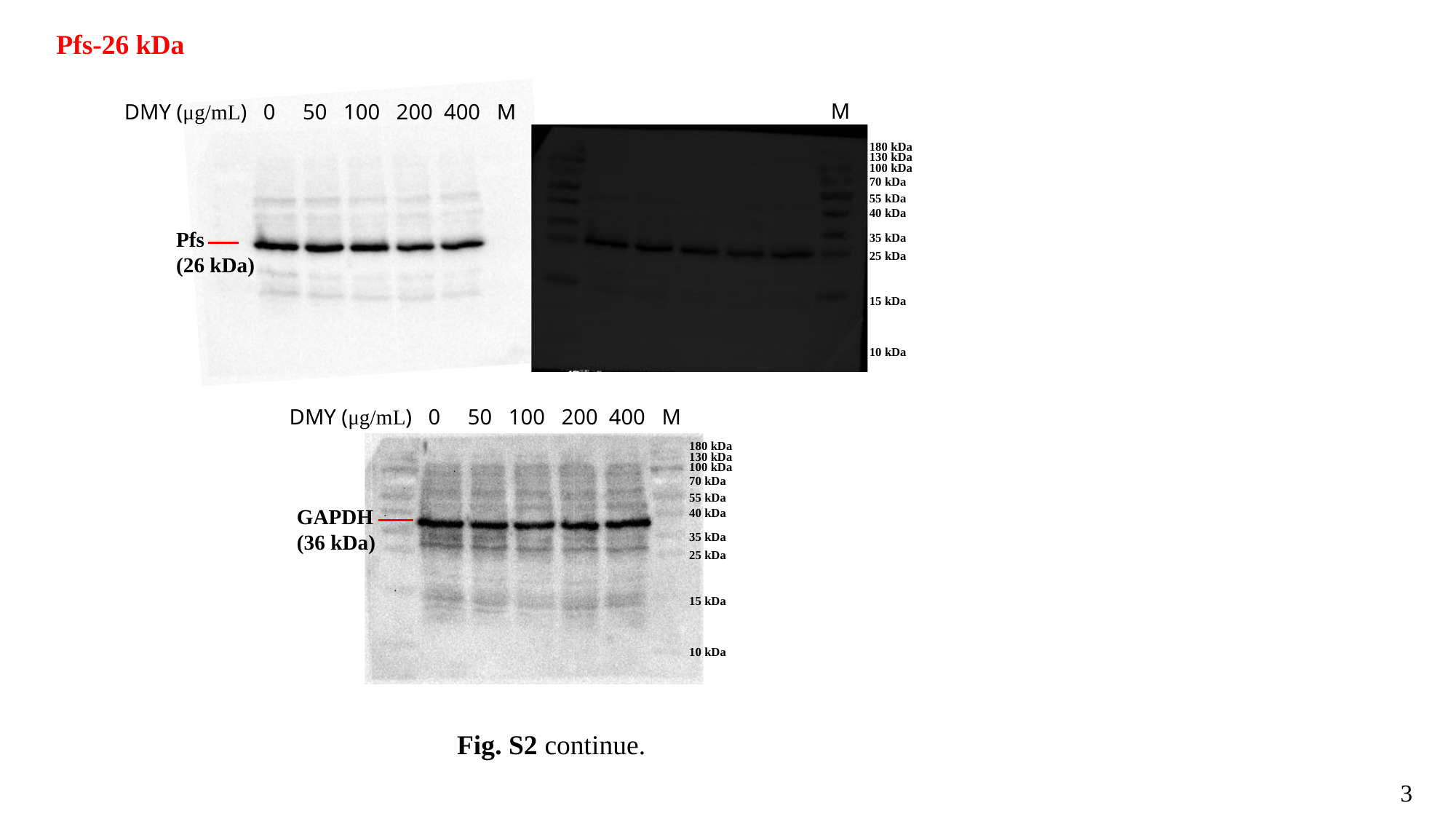

Pfs-26 kDa
M
DMY (μg/mL) 0 50 100 200 400 M
180 kDa
130 kDa
100 kDa
70 kDa
55 kDa
40 kDa
35 kDa
25 kDa
15 kDa
10 kDa
Pfs
(26 kDa)
DMY (μg/mL) 0 50 100 200 400 M
180 kDa
130 kDa
100 kDa
70 kDa
55 kDa
40 kDa
35 kDa
25 kDa
15 kDa
10 kDa
GAPDH
(36 kDa)
 Fig. S2 continue.
3

## Slide 4
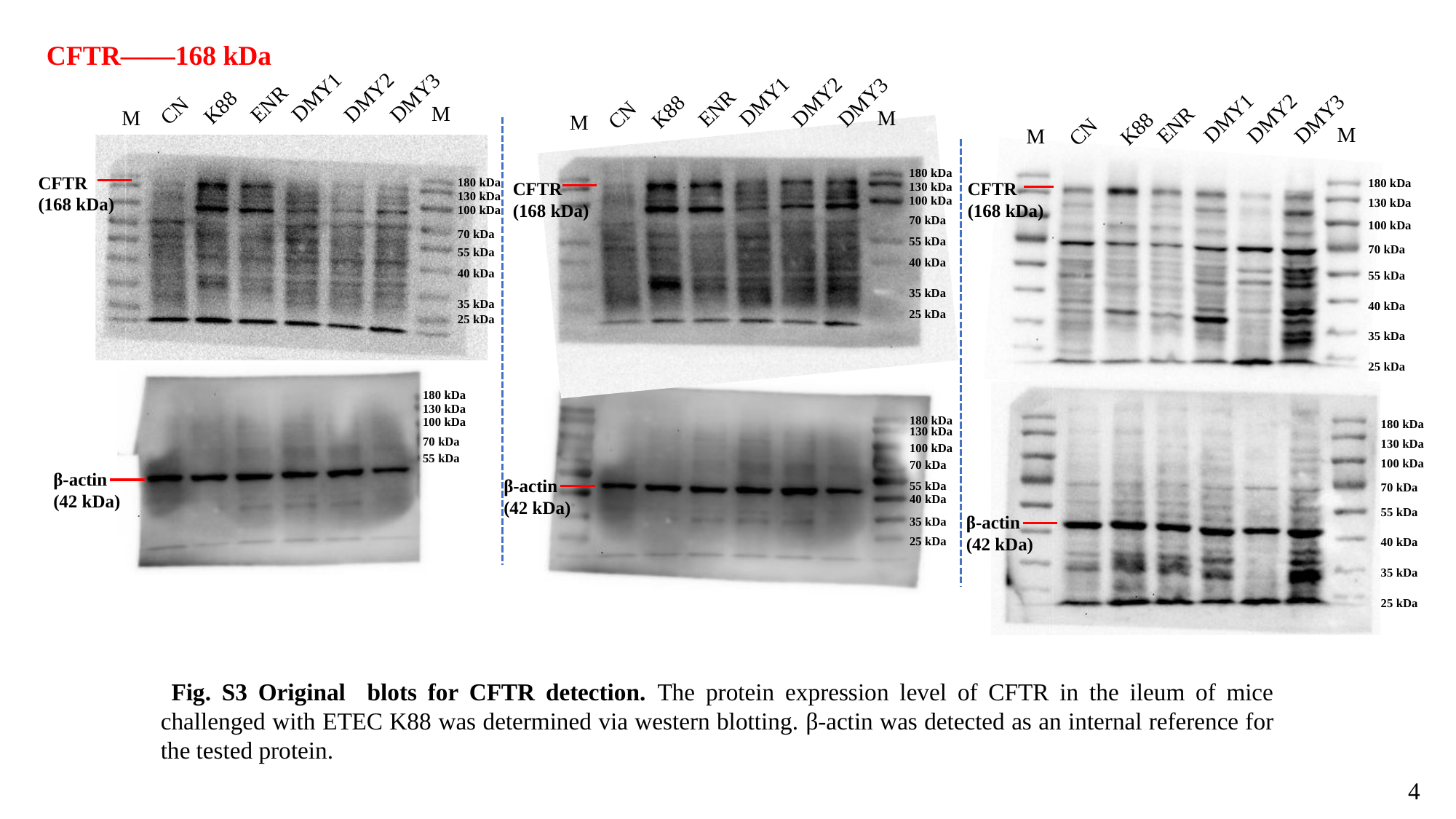

CFTR——168 kDa
DMY1
DMY2
DMY3
DMY1
DMY2
DMY3
ENR
K88
ENR
CN
K88
M
CN
M
DMY1
M
DMY2
DMY3
M
ENR
K88
CN
M
M
180 kDa
130 kDa
100 kDa
70 kDa
55 kDa
40 kDa
35 kDa
25 kDa
CFTR
(168 kDa)
180 kDa
130 kDa
100 kDa
70 kDa
55 kDa
40 kDa
35 kDa
25 kDa
CFTR
(168 kDa)
CFTR
(168 kDa)
180 kDa
130 kDa
100 kDa
70 kDa
55 kDa
40 kDa
35 kDa
25 kDa
180 kDa
130 kDa
100 kDa
70 kDa
55 kDa
180 kDa
130 kDa
100 kDa
70 kDa
55 kDa
40 kDa
35 kDa
25 kDa
180 kDa
130 kDa
100 kDa
70 kDa
55 kDa
40 kDa
35 kDa
25 kDa
β-actin
(42 kDa)
β-actin
(42 kDa)
β-actin
(42 kDa)
 Fig. S3 Original blots for CFTR detection. The protein expression level of CFTR in the ileum of mice challenged with ETEC K88 was determined via western blotting. β-actin was detected as an internal reference for the tested protein.
4

## Slide 5
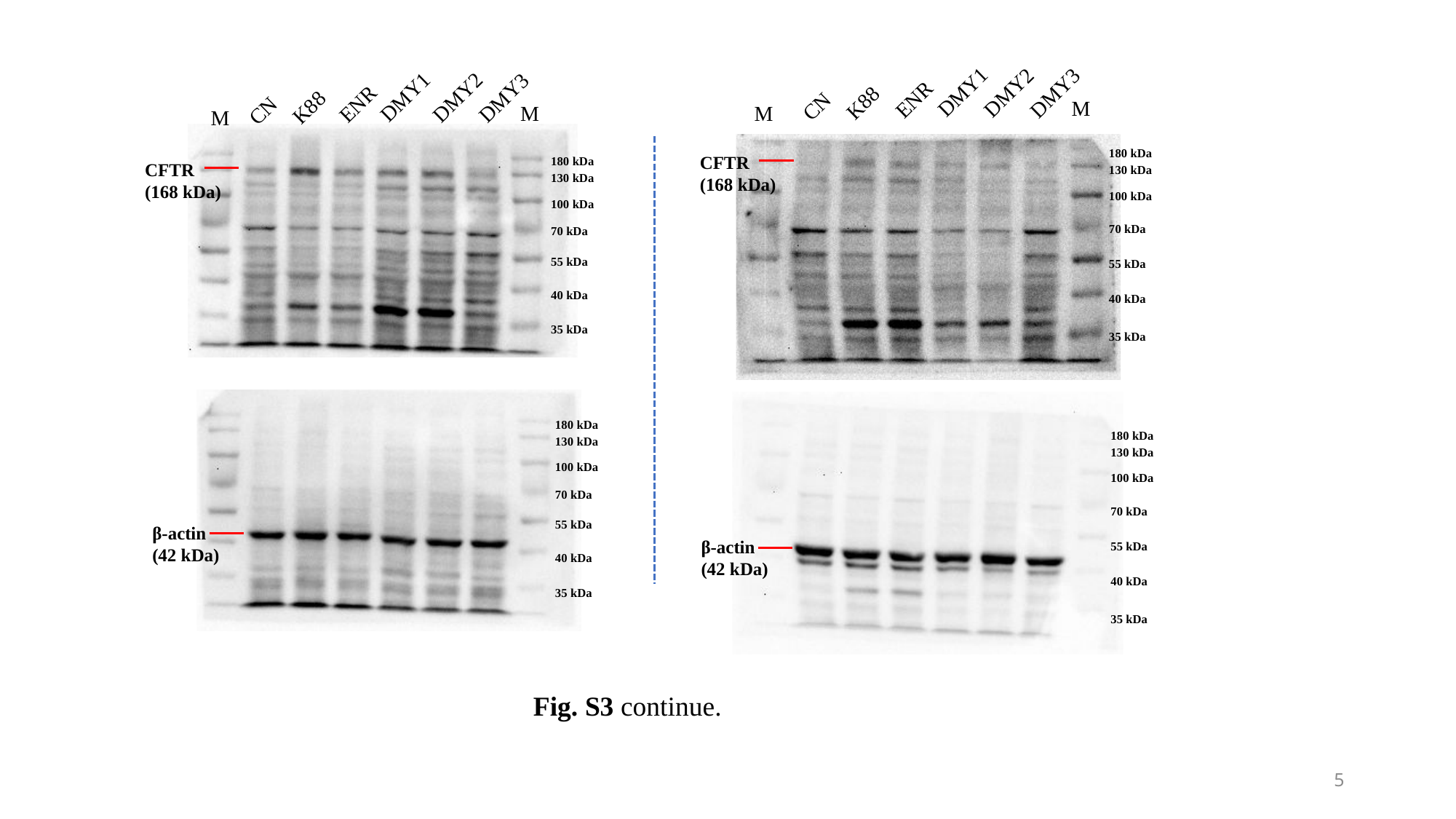

DMY1
DMY2
DMY3
DMY1
DMY2
DMY3
ENR
K88
ENR
CN
K88
M
CN
M
M
M
180 kDa
130 kDa
100 kDa
70 kDa
55 kDa
40 kDa
35 kDa
CFTR
(168 kDa)
180 kDa
130 kDa
100 kDa
70 kDa
55 kDa
40 kDa
35 kDa
CFTR
(168 kDa)
180 kDa
130 kDa
100 kDa
70 kDa
55 kDa
40 kDa
35 kDa
180 kDa
130 kDa
100 kDa
70 kDa
55 kDa
40 kDa
35 kDa
β-actin
(42 kDa)
β-actin
(42 kDa)
Fig. S3 continue.
5

## Slide 6
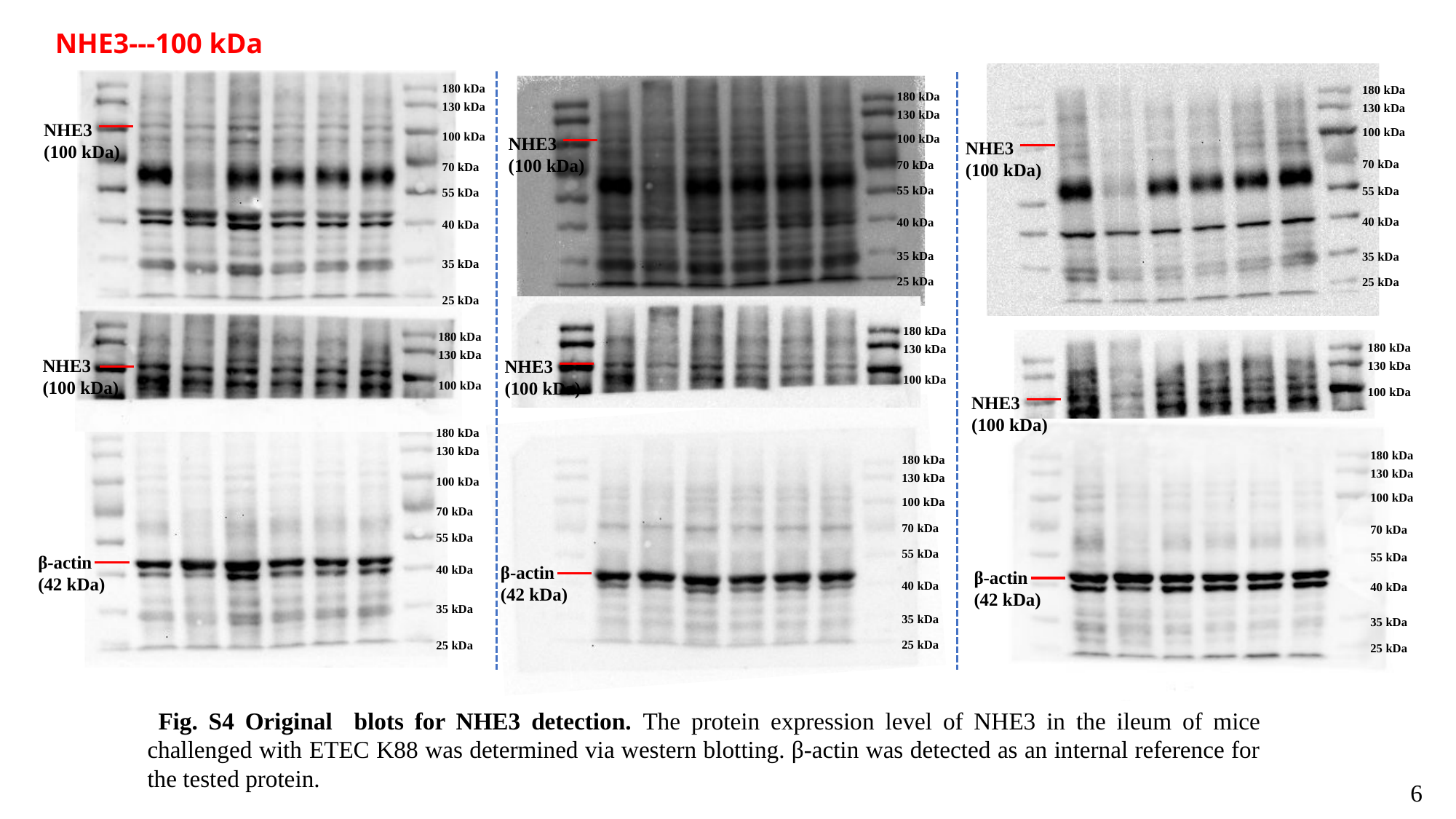

NHE3---100 kDa
180 kDa
130 kDa
100 kDa
70 kDa
55 kDa
40 kDa
35 kDa
25 kDa
180 kDa
130 kDa
100 kDa
70 kDa
55 kDa
40 kDa
35 kDa
25 kDa
180 kDa
130 kDa
100 kDa
70 kDa
55 kDa
40 kDa
35 kDa
25 kDa
NHE3
(100 kDa)
NHE3
(100 kDa)
NHE3
(100 kDa)
180 kDa
130 kDa
100 kDa
180 kDa
130 kDa
100 kDa
180 kDa
130 kDa
100 kDa
NHE3
(100 kDa)
NHE3
(100 kDa)
NHE3
(100 kDa)
180 kDa
130 kDa
100 kDa
70 kDa
55 kDa
40 kDa
35 kDa
25 kDa
180 kDa
130 kDa
100 kDa
70 kDa
55 kDa
40 kDa
35 kDa
25 kDa
180 kDa
130 kDa
100 kDa
70 kDa
55 kDa
40 kDa
35 kDa
25 kDa
β-actin
(42 kDa)
β-actin
(42 kDa)
β-actin
(42 kDa)
 Fig. S4 Original blots for NHE3 detection. The protein expression level of NHE3 in the ileum of mice challenged with ETEC K88 was determined via western blotting. β-actin was detected as an internal reference for the tested protein.
6

## Slide 7
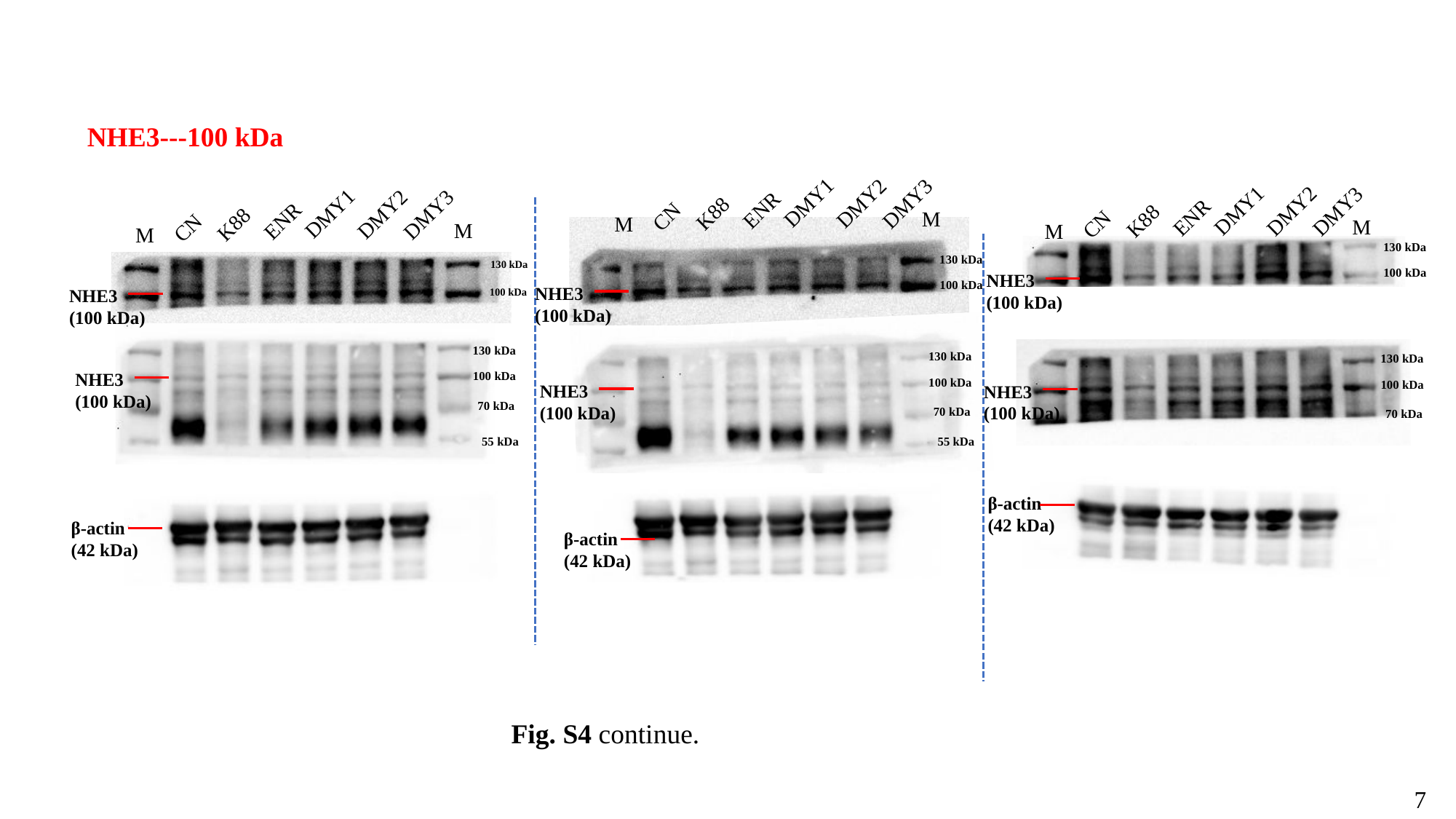

NHE3---100 kDa
DMY1
DMY2
DMY3
ENR
DMY1
DMY2
DMY3
K88
DMY1
DMY2
DMY3
CN
ENR
M
ENR
K88
M
K88
CN
M
CN
M
M
M
130 kDa
130 kDa
130 kDa
100 kDa
NHE3
(100 kDa)
100 kDa
NHE3
(100 kDa)
NHE3
(100 kDa)
100 kDa
130 kDa
130 kDa
130 kDa
NHE3
(100 kDa)
100 kDa
100 kDa
100 kDa
NHE3
(100 kDa)
NHE3
(100 kDa)
70 kDa
70 kDa
70 kDa
55 kDa
55 kDa
β-actin
(42 kDa)
β-actin
(42 kDa)
β-actin
(42 kDa)
 Fig. S4 continue.
7
